# A Rare Location of a Neuroendocrine Tumor

**DOI:** 10.7759/cureus.794

**Published:** 2016-09-21

**Authors:** Gerard Chaaya, Jonathan B Vasquez, Vania Zayat

**Affiliations:** 1 Internal Medicine, UCF College of Medicine; 2 Pathology Department, Orlando VA Medical Center; 3 UCF College of Medicine; 4 Orlando VA Medical Center

**Keywords:** neuroendocrine tumors, duodenum

## Abstract

Neuroendocrine tumors (NETs) arising in the duodenum are rare neoplasms that are often classified as indolent and have a low potential to metastasize. Although rare, multiple reports cite an increasing incidence of duodenal NETs. Symptoms are usually nonspecific and the diagnosis is made via endoscopy. Endoscopic resection is the mainstay of therapy. The prognosis is usually favorable. We describe a case of a duodenal NET that presented with vague symptoms in order to increase the awareness of this rare but increasing in frequency entity.

## Introduction

Neuroendocrine tumors (NETs) are neoplasms of enterochromaffin cell origin that display neurosecretory capacity, which may result in a carcinoid syndrome [1]. Well-differentiated duodenal NET or duodenal carcinoid tumors are rare tumors with only several hundred cases reported in the literature [2]. These tumors may be single and sporadic, functional (such as Zollinger-Ellison syndrome), or familial (such as multiple endocrine neoplasia Type 1 or neurofibromatosis).

## Case presentation

**Figure 1:**
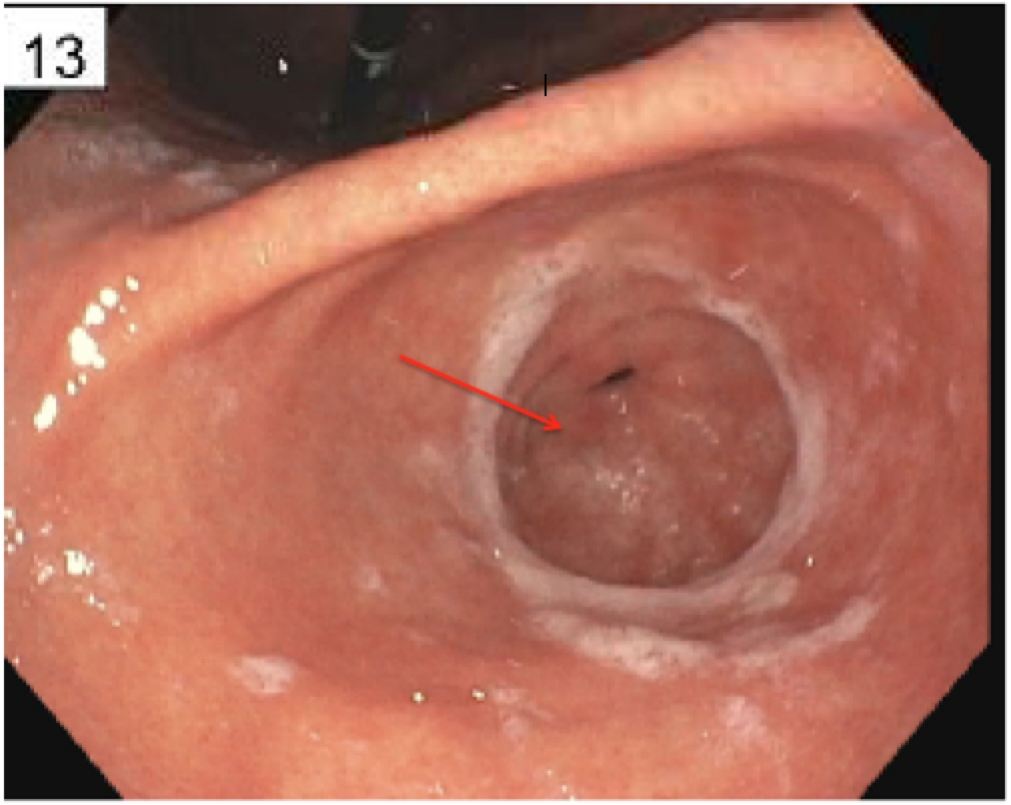
Duodenal Bulb Polyp (red arrow)

**Figure 2:**
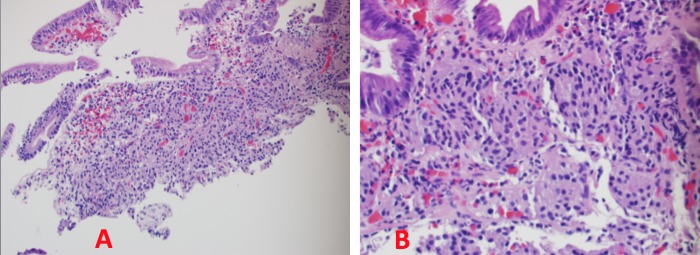
Hematoxylin & Eosin Stain Low power view (A) of the duodenal bulb polyp showing infiltration of tumor cells in the lamina propria. High power view (B) showing an “organoid” arrangement of tumor cells in the lamina propria, along with relatively uniform hyperchromatic round to oval nuclei, coarsely stippled chromatin, and ample cytoplasm.

**Figure 3:**
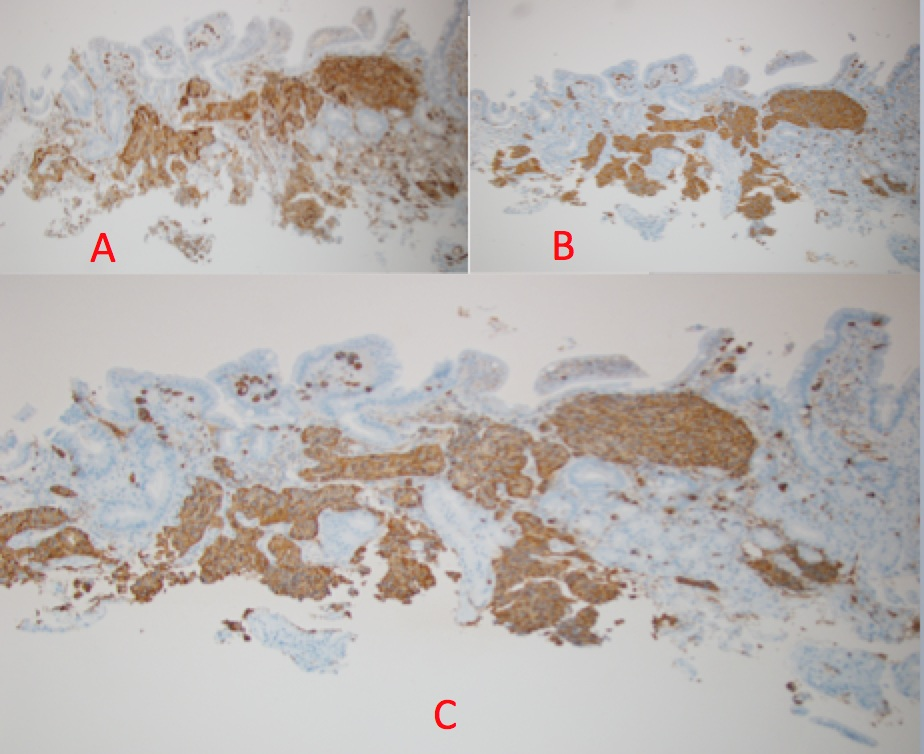
Immunohistochemical Staining A: Positive immunohistochemical stain for CD56 (diffuse cytoplasmic staining). B: Positive immunohistochemical stain for synaptophysin (diffuse cytoplasmic staining). C: Positive immunohistochemical stain for chromogranin (diffuse granular cytoplasmic staining).

A 48-year-old woman, with a past medical and surgical history of hypertension, hyperlipidemia, hypothyroidism, anxiety, depression, and abdominoplasty, presented with a five-month history of intermittent diffuse abdominal pain and bloating. The symptoms were exacerbated by food intake and associated with nausea and alternating diarrhea and constipation. Physical examination was normal, except for a mild diffuse abdominal tenderness. The complete blood count, complete metabolic panel, and the thyroid-stimulating hormone (TSH) were within normal limits. The computed tomography (CT) scan of the abdomen and pelvis with intravenous contrast was unremarkable as well as the colonoscopy. The esophagogastroduodenoscopy (EGD) showed esophagitis in the distal third of the esophagus, chronic gastritis in the body of the stomach and antrum with negative H. pylori on an immunohistochemical stain, and a 0.2 cm duodenal bulb polyp that was resected (Figure 1). The patient consent was given for the procedure. Histopathological examination of the duodenal polyp revealed a small superficial sample of duodenal mucosa with a well-differentiated neuroendocrine tumor in the submucosa (Figure 2). Neuroendocrine marker positivity for CD56 (Figure 3A), synaptophysin (Figure 3B), and chromogranin (Figure 3C) stains supported the diagnosis, which was consistent with a well-differentiated neuroendocrine tumor (NET) of the duodenal bulb. The patient was educated on anti-reflux measures and was started on proton pump inhibitor. A full-body computed tomography (CT) scan showed no evidence of primary or metastatic disease in the brain, chest, abdomen, or pelvis. A video capsule endoscopy did not reveal other small bowel polyps. The patient's symptoms resolved; she will be undergoing surveillance EGD every six months for three years and annually thereafter.

## Discussion

Duodenal NETs comprise 2-3% of all gastrointestinal (GI) endocrine tumors [1]. Although rare, multiple reports cite an increasing incidence of duodenal NETs, with one publication reporting a 400% increase from 1983 to 2010 [3]. This increase might be due to a raised awareness by physicians and a greater frequency of routine endoscopic surveillance. Duodenal NETs can either occur sporadically (75–80%) or can be associated with multiple endocrine neoplasia Type-1 (MEN1) or Zollinger-Ellison syndrome (ZES) [4]. More than 90% of duodenal NETs arise in the first and second part of the duodenum [4]. The symptoms are produced either by virtue of local infiltration resulting in obstructive jaundice, hemorrhage, pancreatitis, abdominal pain, intestinal obstruction, or by clinical features attributed to ectopic hormone release in cases of ZES (gastrin), carcinoid syndrome (serotonin), Cushing’s syndrome (adrenocorticotropic hormone), and acromegaly (growth hormone-releasing hormone) [4]. In the pancreas, these tumors may develop into insulinoma or glucagonoma [4]. Upper gastrointestinal endoscopy is the most sensitive method for detection, but endoscopic ultrasound (EUS) with cytology is helpful to confirm the diagnosis and locally stage the disease [4]. Helical CT can be used to complete the staging of the tumor as well as somatostatin receptor scintigraphy (SRS) using Indium label octreotide analog. Both equally prove to be a sensitive and specific method for staging purposes to stage these tumors.

WHO has classified NETs into well-differentiated NETs; well-differentiated neuroendocrine carcinoma, which has a low malignant potential, and poorly differentiated neuroendocrine carcinomas, representing small cell neuroendocrine carcinomas of high malignancy potential [4]. Histologic examination demonstrates microscopic features typical of endocrine tumors with cells arranged in trabecular, acinar, ribbon, or cribriform patterns. Cytomorphology is uniformly monomorphic and has few to many mitoses; it may demonstrate focal necrosis that is separated by vascularized stroma. On classic silver staining, 75–80% are argyrophilic, argentaffin negative with a positive staining for chromogranin A, neuron-specific enolase, and endocrine cell marker, CD56 (neural cell adhesion molecule) [4].

Endoscopic resection may be considered for non-metastatic duodenal lesions measuring up to 2 cm if the tumor is confined to the mucosa and submucosa on EUS examination [5]. Surgical resection should be performed on tumors > 2 cm. While distant metastases rarely occur with duodenal NETs, lymph node metastases have been reported in tumors < 1 cm. Therefore, surgical resection should be performed in all patients with evidence of lymph node involvement on pretreatment imaging studies. Following surgical resection of a NET, medical therapy may be required for symptom management related to functional tumor syndromes as well as management of progressive metastatic and residual disease [5]. Patients with symptomatic functional NET should be considered for somatostatin (SST) analog (short- or long-acting octreotide), interferon-α therapy alone, or in combination [5]. Biochemical markers (based on the functional status of the underlying tumor) should be followed every three to six months, along with CT or MRI scanning every six months for five years following curative surgical resection. Patients undergoing biologic or cytotoxic therapies should have their clinical response to treatment monitored every three months [5].

The five-year survival for duodenal carcinoid lesions is 60% [5]. Some studies have demonstrated the efficacy of plasma pancreastatin and chromogranin A as a predictor of overall survival for primary duodenal NETs [3].

## Conclusions

Our case illustrates a unique and atypical clinical presentation that has led to the diagnosis of a rare location of NET. Physicians treating patients presenting with non-specific gastrointestinal symptoms should keep this entity in mind. An early diagnosis establishes the tumor stage and predicts the prognosis, which eventually leads to the appropriate therapeutic management.
